# Endoplasmic Reticulum Stress in Spinal Cord Contributes to the Development of Morphine Tolerance

**DOI:** 10.3389/fnmol.2018.00072

**Published:** 2018-03-06

**Authors:** Daiqiang Liu, Yaqun Zhou, Yawen Peng, Peng Su, Zheng Li, Qiaoqiao Xu, Ye Tu, Xuebi Tian, Hui Yang, Zhen Wu, Wei Mei, Feng Gao

**Affiliations:** ^1^Department of Anesthesiology, Tongji Hospital, Tongji Medical College, Huazhong University of Science and Technology, Wuhan, China; ^2^Department of Anesthesiology, Renmin Hospital of Wuhan University, Hubei General Hospital, Wuhan, China

**Keywords:** morphine tolerance, endoplasmic reticulum stress, unfolded protein response, binding immunoglobulin protein, activating transcription factor 6, protein kinase RNA-like ER kinase, inositol-requiring enzyme 1

## Abstract

Morphine tolerance remains an intractable problem, which hinders its prolonged use in clinical practice. Endoplasmic reticulum (ER) stress has been proved to play a fundamental role in the pathogenesis of Alzheimer’s disease, diabetes, atherosclerosis, cancer, etc. In this study, we provide the first direct evidence that ER stress may be a significant driver of morphine tolerance. Binding immunoglobulin protein (BiP), the ER stress marker, was significantly upregulated in neurons in spinal dorsal horn in rats being treated with morphine for 7 days. Additionally, chronic morphine treatment resulted in the activation of three arms of unfolded protein response (UPR): inositol-requiring enzyme 1/X-box binding protein 1 (IRE1/XBP1), protein kinase RNA-like ER kinase/eukaryotic initiation factor 2 subunit alpha (PERK/eIF2α), and activating transcription factor 6 (ATF6). More importantly, inhibiting either one of the three cascades could attenuate the development of morphine tolerance. Taken together, our results suggest that ER stress in spinal cord might contribute to the development of morphine tolerance. These findings implicate a potential clinical strategy for preventing morphine tolerance and may contribute to expanding the morphine usage in clinic.

## Introduction

Morphine is a classical agonist of μ opioid receptor, which has been widely used for the treatment of acute and chronic pain due to its potent analgesic effect. Repeated administration of morphine always lead to the drug tolerance, and patients may need higher doses of drug to achieve comparable pain relief (King et al., 2005; Williams et al., 2013). Considerable advance has been made in the mechanisms underlying morphine tolerance, including desensitization and internalization of opioid receptor (Narita et al., 2006; Martini and Whistler, 2007), heterodimers of G protein-coupled receptors (Pasternak and Pan, 2011; Szentirmay et al., 2013), involvement of chemokines (Ye et al., 2014; Guo and Gao, 2015; Guo et al., 2017; Wang et al., 2017), etc. However, detailed mechanisms are still far from clear and few strategies are available for the management of morphine tolerance for now. Therefore, further studies are needed to explore novel therapeutic targets for morphine tolerance.

Endoplasmic reticulum (ER) stress is a complex pathophysiological process, which occurs due to the disturbances of ER homeostasis such as glucose deprivation, accumulation of unfolded or misfolded proteins, and oxidative stress (Xu et al., 2005; Bettigole and Glimcher, 2015). In an attempt to restore the intracellular homeostasis, ER stress could lead to the activation of UPR, which is mediated by three ER stress sensors and related signaling pathways: IRE1, PERK and ATF6 (Ron and Walter, 2007; Mori, 2015). Under physiological conditions, ER stress sensors are kept in the inactive state through being bound by chaperone BiP (Bertolotti et al., 2000). However, BiP could dissociate from the sensors when ER stress occurs, resulting in the activation of UPR. ER stress has been reported to play a fundamental role in the pathogenesis of many diseases including neurodegenerative disorders (Doyle et al., 2011), metabolic syndrome (Piperi et al., 2012), cardiovascular diseases (Liu et al., 2016) and cancer (Wang et al., 2014). And it might also be involved in the induction and maintenance of neuropathic pain (Inceoglu et al., 2015; Zhang et al., 2015) and inflammatory pain (Yang E.S. et al., 2014). Recent studies demonstrated that chronic morphine exposure could induce the oxidative stress in spinal neural cells (Cai et al., 2016; Lauro et al., 2016), and BiP mutation in mice could attenuate the development of morphine tolerance (Dobashi et al., 2010), which indicate the pivotal role of BiP in the mechanism of morphine tolerance. Besides, currently evidences have proved the involvement of ER stress in the pathogenesis of SCI (Penas et al., 2007; Ohri et al., 2011). However, whether ER stress is involved in the mechanism of morphine tolerance remains unknown. In this study, we sought to investigate the expressions of BiP and ER stress-related signaling pathways in spinal cord, and evaluate the effects of inhibiting spinal ER stress sensors during the development of morphine tolerance, to explore the potential role of ER stress in morphine tolerance.

## Materials and Methods

### Animals

Adult male Sprague-Dawley rats weighing 220–250 g, were purchased from Laboratory Animal Center, Tongji Medical College, Huazhong University of Science and Technology. Animals were housed individually under controlled conditions (22 ± 0.5°C, relative humidity 40–60%, standard 12: 12 h light: dark cycles, food and water *ad libitum*). All experimental procedures and protocols were reviewed and approved by Experimental Animal Care and Use Committee of Tongji Medical College, Huazhong University of Science and Technology, and carried out in accordance with the National Institutes of Health Guidelines for the Care and Use of Laboratory Animals.

### Intrathecal Catheterization

For drug administration, intrathecal (i.t.) catheters were implanted using a lumbar approach, as described previously (Zhou et al., 2017). Briefly, animals were anesthetized with intraperitoneal injection (i.p.) of pentobarbital sodium (60 mg/kg). The lumbar region of rat was shaved. A sterile polyethylene catheter (PE-10; outer diameter 0.5 mm, inner diameter 0.3 mm; Anilab Software & Instruments, Ningbo, China) was inserted into subarachnoid cavity between L4 and L5 vertebrae. The catheter was subcutaneously tunneled, externalized, and fixed to the back of neck. Wounds were sutured after disinfection with 75% (v/v) ethanol. Correct intrathecal placement of catheter was verified by a temporary motor block of both hind limbs after intrathecal injection of 10 μL of 2% lidocaine. Rats were housed individually after surgery and allowed a 7-day recovery period before the following experiments. Rats with hind limb paralysis or paresis after surgery were excluded and euthanized with overdose of pentobarbital sodium.

### Drug Administration

The drugs used in this study were prepared as follows. Morphine hydrochloride (Shenyang First Pharmaceutical Factory, China) was diluted in saline (Northeast Pharmaceutical Group, China). Specific IRE1α endonuclease inhibitor STF-083010 (Selleckchem, Houston, TX, United States) and selective PERK inhibitor GSK2606414 (Selleckchem, Houston, TX, United States) were dissolved in 100% dimethyl sulfoxide (DMSO, Sigma, St. Louis, MO, United States), respectively. STF-083010 (10 or 50 μg), GSK2606414 (10 or 100 μg) or vehicle solution was intrathecal injected 30 min before morphine administration in a volume of 10 μL, respectively, followed by 10 μL of saline to flush the catheter. The doses of STF-083010 and GSK2606414 used in this study were determined according to our preliminary results.

### Morphine Tolerance

Morphine tolerance was induced by intrathecal administration of morphine (10 μg, twice daily) for 7 days, as described previously (Guo et al., 2016). Rats in the control group received an equivalent volume of saline at the same time points. The development of morphine tolerance was assessed by behavioral tests on days 1, 3, 5, and 7 (Peng et al., 2017).

### Behavioral Assessment

Thermal pain thresholds in rats were measured by a tail-flick latency test before drug administration and at 30 min after morphine administration on days 1, 3, 5, and 7 (Cui et al., 2008). Briefly, rat was placed in a plastic container. The body of rat was restrained and one-third to the tip of tail was immersed into water which was maintained at 50 ± 0.2°C. A positive response was defined as the rapid removal of tail from hot water. A cutoff time of 15 s was determined to prevent tail damage. The test was repeated three times with an interval of 5 min and the mean of three tests was considered as the final latency. The percentage of maximal possible antinociceptive effect (%MPE) was calculated by comparing the test latency before (baseline, BL) and after drug injection (TL) using the following equation: %MPE = [(TL - BL)/(cutoff time - BL)] × 100. All the behavioral tests were performed by an investigator who was blinded to the experimental design.

### Quantitative Real-Time Polymerase Chain Reaction (qRT-PCR)

Rats were deeply anesthetized with pentobarbital sodium (60 mg/kg, i.p.) and L3-L5 spinal cord segments were quickly removed. Total RNA of spinal cord tissue from each group was extracted using Trizol Reagent (Invitrogen, Carlsbad, CA, United States) according to the manufacturer’s instructions and then synthesized to cDNA by reverse transcription. StepOne Real-Time PCR System (Applied Biosystems, Carlsbad, CA, United States) was used to conduct the qRT-PCR. One microgram of total RNA from each sample was added into 20 μL reactive solution of reverse transcription, respectively. The specific primer sequences for target genes in this study were designed and synthesized by Takara Biomedical Technology (Kyoto, Japan) and listed in **Table 1**. Housekeeping gene GAPDH was used as an internal control. Relative quantification of mRNA was performed by 2^-ΔΔCt^ method.

**Table 1:** Primers for Real-time PCR.

Name	Primer	Sequence	Size
Rat GAPDH	Forward	5′- ACAGCAACAGGGTGGTGGAC -3′	253 bp
	Reverse	5′- TTTGAGGGTGCAGCGAACTT -3′	
Rat ATF6	Forward	5′- AGCCCCTCATTAACACGACA -3′	158 bp
	Reverse	5′- AGAATTCGAGCCCTGTTCCA -3′	
Rat IRE1	Forward	5′- CGGGAGAGCTGTGGTTAAGA -3′	244 bp
	Reverse	5′- TCGGTAGGTGTGAGAGAGGA -3′	
Rat XBP1u	Forward	5′- AGACTACGTGCGCCTCTGCA -3′	170 bp
	Reverse	5′- AGACTCTGGGGAAGGACATT -3′	
Rat XBP1s	Forward	5′- GCTTTCATCCAGCCATTGTCT -3′	150 bp
	Reverse	5′- AGTTCGTTGGCAAAAGTGTC -3′	
Rat PERK	Forward	5′- AGTCGGTCTTTCTCAGTGGG -3′	160 bp
	Reverse	5′- CCATGTCGCAATCTGTCAGG -3′	
Rat eIF2α	Forward	5′- CTCGCAACGCAGCATTCTAT -3′	187 bp
	Reverse	5′- GCACACGTGGCTGTTAAGAT -3′	
Rat BiP	Forward	5′- GAACCAACTCACGTCCAACC -3′	247 bp
	Reverse	5′-CTTTCCCAAATACGCCTCGG -3′	

### Western Blots

Rats were deeply anesthetized with sodium pentobarbital (60 mg/kg, i.p.) and L3–L5 spinal cord segments were quickly removed. Total proteins of spinal cord tissue from each group were extracted using RIPA lysis buffer combined with a mixture of proteinase inhibitors according to the manufacturer’s instruction (Boster, Wuhan, China). The protein concentration of supernatants was measured by using bicinchoninic acid assay. 50 μg protein from each sample was loaded and separated on 10% SDS-PAGE. Electrophoresis was conducted at 60 V constant voltage for stacking gel and 100 V for separating gel. The proteins were subsequently electro-transferred (200 mA, 60–90 min) to a PVDF membrane (IPVH00010, Millipore, Bellerica, MA, United States). The membranes were blocked with 5% bovine serum albumin for 1 h at room temperature (RT) and then incubated overnight at 4°C with the following primary antibodies: rabbit anti-BiP antibody (1:4000; ab21685; Abcam, Cambridge, MA, United States), rabbit anti-IRE1 antibody (1:4000; ab37073; Abcam, Cambridge, MA, United States), rabbit anti-XBP1 antibody (1:2000; A1731; ABclonal, Wuhan, China), rabbit anti-PERK (C33E10) antibody (1:1000; #3192; CST, Beverly, MA, United States), rabbit anti-phospho-PERK (Thr980) (16F8) antibody (1:1000; #3179; CST, Beverly, MA, United States), rabbit anti-eIF2α (D7D3) antibody (1:2000; #5324; CST, Beverly, MA, United States), rabbit anti-phospho-eIF2α (Ser51)(D9G8) antibody (1:1000; #3398; CST, Beverly, MA, United States), rabbit anti-ATF6 antibody (1:1000; ab203119; Abcam, Cambridge, MA, United States) and rabbit anti-GAPDH antibody (1:5000; AS1039; Aspen, Wuhan, China). After being thoroughly washed, the membranes were incubated with HRP – conjugated goat anti-rabbit IgG (1:5000, Aspen, Wuhan, China) for 2 h at RT. Finally, proteins were detected by SuperLumia ECL Plus HRP Substrate Kit (K22030, Abbkine, Wuhan, China) and a computerized image analysis system (Bio-Rad, ChemiDoc XRS+, United States). Image Lab software (Bio-Rad Laboratories) was used to quantify the intensity of protein blots, which were normalized to loading control GAPDH and expressed as the fold of control. The blot density of control group was set as 1.

### Immunofluorescent Staining

After being treated with morphine or saline for 7 days, rats were deeply anesthetized with sodium pentobarbital (60 mg/kg, i.p.) and perfused intracardially with saline followed by 4% ice-cold PFA in 0.1 M phosphate buffer saline (PBS). After perfusion, L3–L5 spinal cord segments were quickly removed and post-fixed in 4% PFA for 4 h and subsequently dehydrated in 30% sucrose solution for 2 days at 4°C. Transverse spinal sections (20 μm) were cut in a cryostat (CM1900, Leica, Germany), mounted on poly-lysine-coated slides, and stored at -80°C until use.

#### Single Immunostainings

The sections were penetrated with 0.3% Triton-X-100 for 15 min and blocked with 10% donkey serum for 1 h at RT. The sections were then incubated overnight at 4°C with the following primary antibodies: rabbit anti-BiP antibody (1:500; ab21685; Abcam, Cambridge, MA, United States), rabbit anti-IRE1 antibody (1:50; ab37073; Abcam, Cambridge, MA, United States), rabbit anti-XBP1 antibody (1:50; A1731; ABclonal, Wuhan, China), rabbit anti-PERK (phospho T981) antibody (1:50; YP1055; ImmunoWay, Plano, TX, United States), rabbit anti-phospho-eIF2α (Ser51) (D9G8) antibody (1:50; #3398; CST, Beverly, MA, United States), rabbit anti-ATF6 antibody (1:50; ab203119; Abcam, Cambridge, MA, United States). Then, the sections were incubated with Alexa Fluor 488-labeled donkey anti-rabbit secondary antibody (1:300; A-21206; Invitrogen, Carlsbad, CA, United States) for 2 h at RT and washed with PBS. Sections were rinsed, mounted, and cover-slipped with 50% glycerol. Images were captured using a fluorescence microscope (DM2500, Leica, Germany). BiP/IRE1/XBP1/p-PERK/p-eIF2α/ATF6-immunolabeled surface areas were measured in laminae I–IV of spinal cord dorsal horn using Image Pro Plus 4 software (Media Cybernetics, Maryland, MD, United States). Quantification of immunoreactivity was accomplished by calculating the percentages of immunostaining [(positive immunofluorescent surface area)/(total measured picture area) × 100]. Three rats of each group were used for statistical analysis. All the image analyses were performed by an investigator who was blinded to the experimental design.

#### Double Immunofluorescent Staining

For double immunofluorescence, the sections were incubated with a mixture of two primary antibodies followed by a mixture of Alexa 488-conjugated and Alexa 594-conjugated secondary antibodies (1:300; A-21207; Invitrogen, Carlsbad, CA, United States). Specifically, to identify the cell types expressing BiP, IRE1, XBP1, PERK (phospho T981), Phospho-eIF2α (Ser51) or ATF6, each of the primary antibodies was mixed with the antibody of neuronal marker neuronal nuclei (NeuN; 1:50; MAB377; Millipore, Billerica, MA, United States), astrocytic marker GFAP (1:200; #3670; CST, Beverly, MA, United States), or microglial marker Iba1 (1:100; ab5076; Abcam, Cambridge, MA, United States), respectively. Images were captured with a fluorescence microscope (DM2500, Leica, Germany). Non-specific staining was determined by omitting the primary antibodies.

### Preparation of ATF6 RNAi-Lentivirus

According to the previous literatures (Seo et al., 2008, 2010), recombinant shRNA lentiviral vectors targeting rat ATF6 gene (ATF6 RNAi-LV) and non-specific control lentivirus (NC-LV) were designed by Genechem (Shanghai, China). Briefly, ATF6 (5′-CCATTGTGTTACCAGCAAT-3′) or non-specific control shRNA oligonucleotides (5′-TTCTCCGAACGTGTCACGT-3′) were inserted into lentivirus transfer vector (GV248-LV, GeneChem Co. Ltd., Shanghai, China) under the control of U6 promoter. Lentiviruses were acquired from triple-infected 293T cells with approximately 80% confluence. The lentiviral vector backbone was hU6-MCS-Ubiquitin-EGFP-IRES-puromycin. The pellets were suspended in DMEM, aliquoted, and stored at -80°C. The viral titer for stock was 3.0 × 10^8^ TU/mL medium.

### Stereotaxic Injection of RNAi-Lentivirus Into Spinal Dorsal Horn

Stereotaxic injection of lentiviral vectors into spinal dorsal horn was conducted 3 days before intrathecal catheterization according to the methods described previously (Fu et al., 2016). Briefly, rats were anesthetized with pentobarbital sodium (60 mg/kg, i.p.). The lumbar region of rat was shaved. A longitudinal skin incision was performed along the midline of lumbar spine to cautiously expose the L1 vertebrae. Two holes of 1 mm diameter were carefully drilled on the left and right of vertebrae (0.5 mm from the midline). Then, rats were placed on a stereotaxic instrument. The tip of a Hamilton microsyringe was insert into spinal dorsal horn of rats through each hole on the vertebrae at a depth of 0.8 mm. 2 μL of ATF6 RNAi-LV or NC-LV solution was administered through each hole at a rate of 0.5 μL/min, respectively. The needle was left in spinal cord for at least 2 min before removed. After surgery, rats were kept in an insulation can for 30 min for complete recovery from anesthesia.

### Statistical Analyses

Animal sample size for behavioral experiment was decided by power analysis using SSize2021 software (National University of Singapore, Singapore) (version 2). With anticipated population proportion P1 = 0.95, P2 = 0.05, significance level 0.05 and power of test 0.09, the sample size was estimated to be four per group. All data were presented as mean ± SEM and analyzed using GraphPad Prism 5 (GraphPad Software Inc.). Behavioral test was analyzed by two-way repeated measure ANOVA (treatment group × time) to detect overall differences among treatment groups followed by Bonferroni’s test to detect the changes of %MPE after drug injection over time. The results of qRT-PCR and western blots were analyzed by one-way ANOVA followed by Bonferroni *post hoc* test. Individual comparisons were conducted with unpaired *t*-test. *p* < 0.05 was considered statistically significant.

## Results

### Chronic Morphine Treatment Induced Drug Tolerance and Increased BiP Expression in Spinal Cord

**FIGURE 1:**
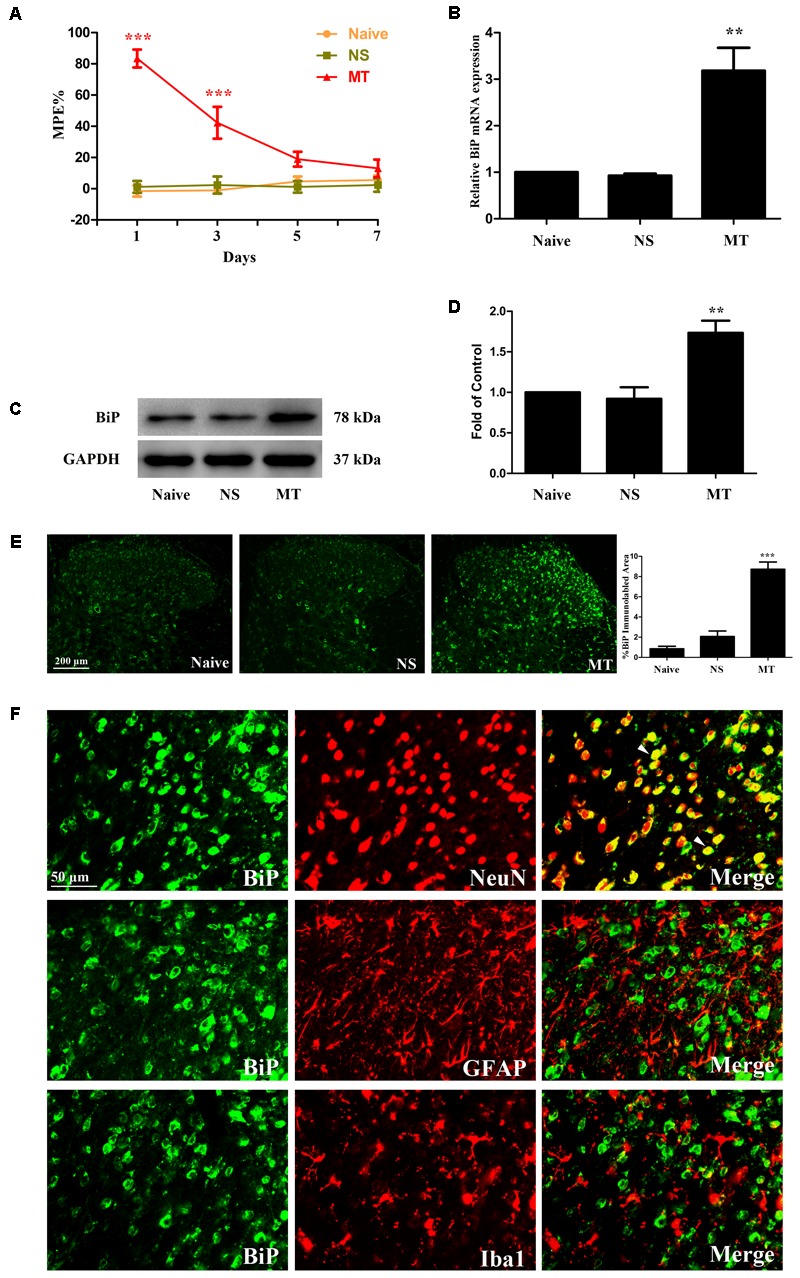
Expression of BiP in lumbar spinal cord. **(A)** Thermal pain threshold of rats was assessed using the percentage of maximal possible antinociceptive effect (%MPE) according to the tail-flick latency of rats. The %MPE in rats received morphine (10 μg, twice daily, intrathecally) on days 5 and 7 were dramatically decreased compared with the baseline on day 1. (^∗∗∗^*p* < 0.001 compared with naive rats, *n* = 6 in each group). **(B–D)** The expression of BiP was significantly increased in morphine-tolerant rats measured by real-time PCR and western blots, respectively. (^∗∗^*p* < 0.01 compared with naive rats, *n* = 4 in each group). **(E)** Immunostaining of BiP in spinal dorsal horn. BiP expression was significantly increased in morphine-tolerant rats compared with those in naïve rats. (^∗∗∗^*p* < 0.001 compared with naive rats, *n* = 3 in each group, scale bar: 200 μm). **(F)** Double immunostaining of BiP and cell-specific markers in morphine-tolerant rats. BiP was co-localized with NeuN (indicated by arrows). Scale bar: 50 μm. BiP, binding immunoglobulin protein; NS, normal saline; MT, morphine tolerance.

Rats were intrathecal administered with morphine (10 μg/5 μL) or saline (5 μL) twice daily for consecutive 7 days. Behavioral tests were conducted before drug administration and at 30 min after the last drug administration on days 1, 3, 5, and 7. As shown in **Figure 1A**. This resulted in reliable interaction between morphine and time (*F*_3,45_ = 12.81), morphine and saline (*F*_2,45_ = 49.91), %MPE in rats received morphine were significantly higher than those in saline-treated rats on days 1 and 3 (*p* < 0.001). There was no statistically significant difference in %MPE levels between morphine-treated and saline-treated rats on days 5 and 7 (*p* > 0.05), indicating that chronic morphine tolerance had been successfully established.

To investigate the involvement of ER stress in morphine tolerance, we first examined the spinal expression of BiP, a marker of ER stress. On day 7, increased expressions of BiP mRNA (*F*_2,11_ = 20.01) and protein (*F*_2,11_ = 14.21) were detected in spinal cord of rats treated with morphine (**Figures 1B–D**, *p* < 0.01 compared to naïve and saline-treated rats), indicating that ER stress might be induced by chronic morphine treatment. Next, we examined the distribution and cellular localization of BiP in spinal cord. The results showed that BiP was extensively expressed in spinal dorsal horn (**Figure 1E**). The immunoreactivity of BiP in morphine-tolerant rats was significantly higher than those in naïve and saline-treated rats (*F*_2,8_ = 61.49, *p* < 0.001). And BiP was found to be co-localized with neuronal marker NeuN, not astrocytic marker GFAP or microglial marker Iba1 (**Figure 1F**). These indicate that upregulation of BiP expression in neurons in spinal dorsal horn might be involved in the development of morphine tolerance.

### Spinal IRE1/XBP1 Cascade Contributed to the Development of Morphine Tolerance

**FIGURE 2:**
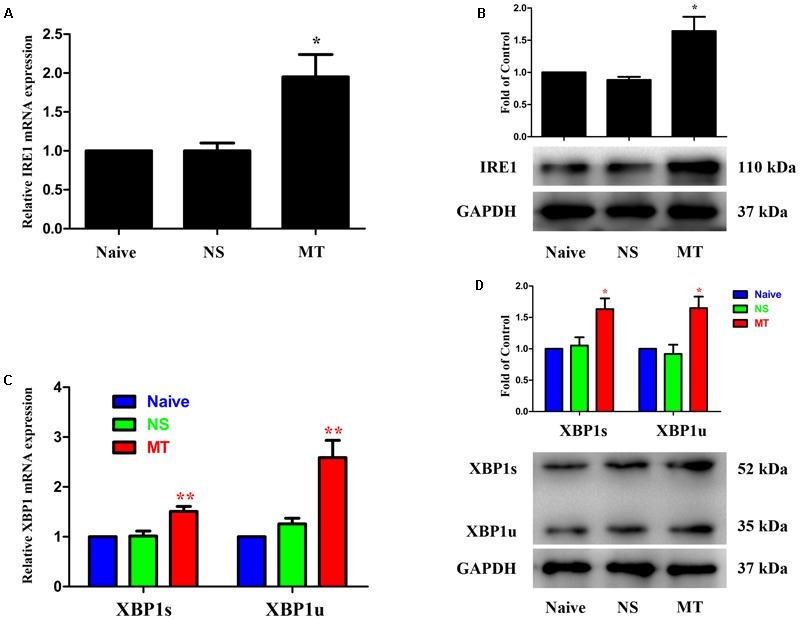
Expressions of IRE1/XBP1 cascade in spinal cord. The expressions of IRE1 **(A,B)**, XBP1s and XBP1u **(C,D)** were significantly increased in morphine-tolerant rats measured by real-time PCR and western blots, respectively. (^∗^*p* < 0.05, ^∗∗^*p* < 0.01 compared with naive rats, *n* = 4 in each group) IRE1: inositol-requiring enzyme 1. XBP1, X-box binding protein 1; XBP1s, spliced variant of XBP1; XBP1u, unspliced XBP1; NS, normal saline; MT, morphine tolerance.

**FIGURE 3:**
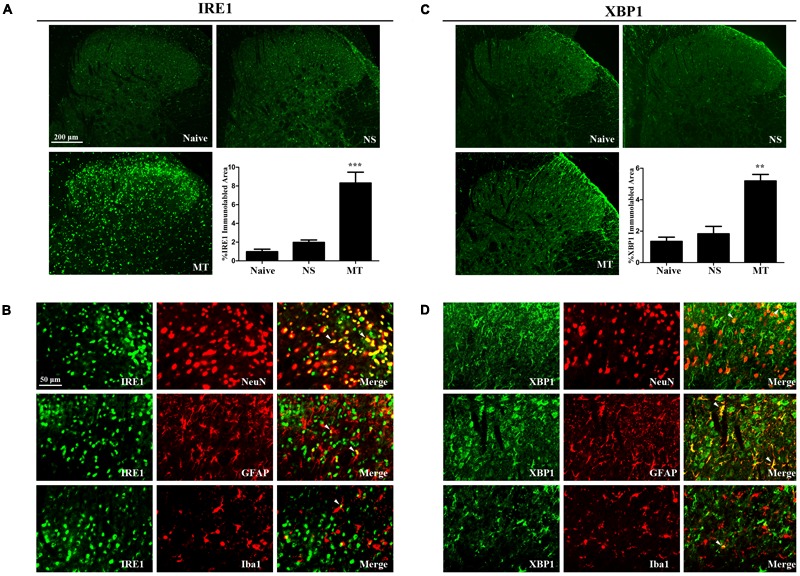
Distributions and cellular localizations of IRE1 and XBP1 in spinal cord. **(A,C)** Immunostaining of IRE1 and XBP1 in spinal dorsal horn. IRE1 and XBP1 were extensively expressed in spinal dorsal horn. The immunoreactivity of IRE1 and XBP1 were significantly increased in morphine-tolerant rats. (^∗∗^*p* < 0.01, ^∗∗∗^*p* < 0.001 compared with naive rats, *n* = 3 in each group, scale bar: 200 μm). **(B,D)** Double immunostaining of IRE1 or XBP1 and cell-specific markers in morphine-tolerant rats. IRE1 and XBP1 were both mainly co-localized with NeuN and GFAP, and a minority with Iba1 (indicated by arrows). Scale bar: 50 μm. IRE1, inositol-requiring enzyme 1; XBP1, X-box binding protein 1; NS, normal saline; MT, morphine tolerance.

Three ER stress sensors (IRE1, PERK, and ATF6) implement the UPR pathways. We first investigated the expressions of IRE1/XBP1 cascade during the development of morphine tolerance. The expressions of IRE1, XBP1s, XBP1u were examined on day 7 of morphine administration. As shown in **Figure 2**, both mRNA and protein levels of IRE1, XBP1s, and XBP1u were significantly increased in morphine-treated rats compared with naïve and saline-treated rats (*F*_2,11_ = 9.742 for mRNA of IRE1, *F*_2,11_ = 13.14 for mRNA of XBP1s, *F*_2,11_ = 16.56 for mRNA of XBP1u, *F*_2,11_ = 9.709 for protein of IRE1, *F*_2,11_ = 7.884 for protein of XBP1s, *F*_2,11_ = 8.723 for protein of XBP1u, *p* < 0.05), as well as the immunoreactivity of IRE1 (**Figure 3A**, *F*_2,8_ = 31.84, *p* < 0.001) and XBP1 (**Figure 3C**, *F*_2,8_ = 28.07, *p* < 0.01) in spinal dorsal horn. To determine the cellular localization of IRE1 and XBP1 in spinal cord, double immunofluorescent staining was performed in morphine-treated rats. The results showed that IRE1 and XBP1 were mainly co-localized with NeuN and GFAP, and a minority with Iba1 in spinal dorsal horn (**Figures 3B,D**). These results indicate that chronic morphine treatment could result in the activation of IRE1/XBP1 cascade mainly in neurons and astrocytes in spinal cord.

**FIGURE 4:**
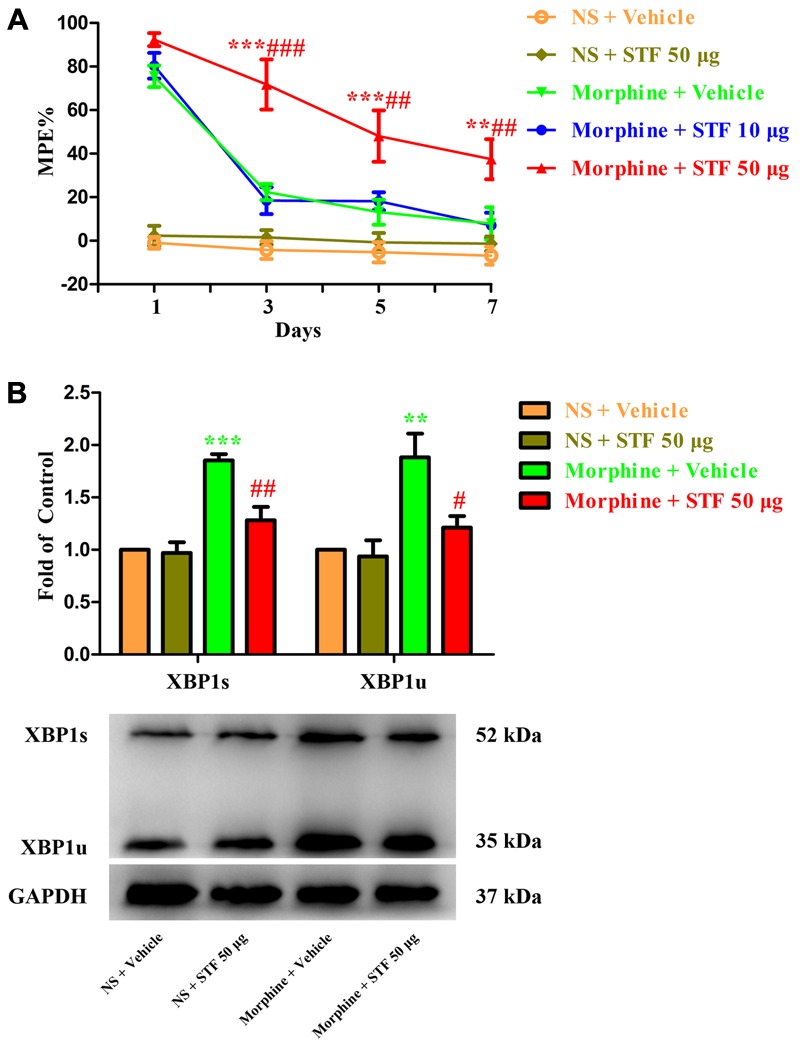
Effect of IRE1 inhibitor on the development of morphine tolerance. Specific IRE1α endonuclease inhibitor STF-083010 (10 μg or 50 μg) was intrathecal injected 30 min before morphine treatment for 7 days, respectively. **(A)** Pretreatment with 50 μg STF-083010 could attenuate the development of morphine tolerance. (^∗∗^*p* < 0.01, ^∗∗∗^*p* < 0.001 compared with morphine + vehicle group; ^##^*p* < 0.01, ^###^*p* < 0.001 compared with morphine + STF 10 μg group, *n* = 5 in each group). **(B)** Pretreatment with 50 μg STF-083010 could inhibit the up-regulation of XBP1s and XBP1u induced by chronic morphine treatment measured by western blots. (^∗∗^*p* < 0.01, ^∗∗∗^*p* < 0.001 compared with NS + vehicle group; ^#^*p* < 0.05, ^##^*p* < 0.01 compared with morphine + vehicle group, *n* = 4 in each group). %MPE, percentage of maximal possible antinociceptive effect; NS, normal saline; STF, STF-083010; XBP1, X-box binding protein 1; XBP1s, spliced variant of XBP1; XBP1u, unspliced XBP1.

To further investigate the role of spinal IRE1/XBP1 cascade in the development of morphine tolerance, a specific IRE1α endonuclease inhibitor STF-083010 was intrathecal injected 30 min before morphine administration. As shown in **Figure 4A**, a single dose of STF-083010 (10 μg or 50 μg) did not affect the antinociceptive effect of morphine on day 1 of morphine administration (*p* > 0.05), but consecutive treatments with STF-083010 (50 μg, but not 10 μg) could significantly attenuate the development of morphine tolerance from days 3 to 7 (*F*_1,24_ = 0.046, for 10 μg STF-083010, *p* > 0.05; *F*_1,24_ = 14.03, for 50 μg STF-083010, *p* < 0.05). Moreover, the increased expressions of XBP1s and XBP1u induced by chronic morphine treatment could be inhibited by STF-083010 pretreatment (**Figure 4B**, *F*_3,15_ = 22.35, for protein of XBP1s, *F*_3,15_ = 8.566 for protein of XBP1u, *p* < 0.05). Taken together, these results suggest that activation of spinal IRE1/XBP1 cascade in neurons and astrocytes might be involved in the development of morphine tolerance.

### Spinal PERK/eIF2α Cascade Contributed to the Development of Morphine Tolerance

**FIGURE 5:**
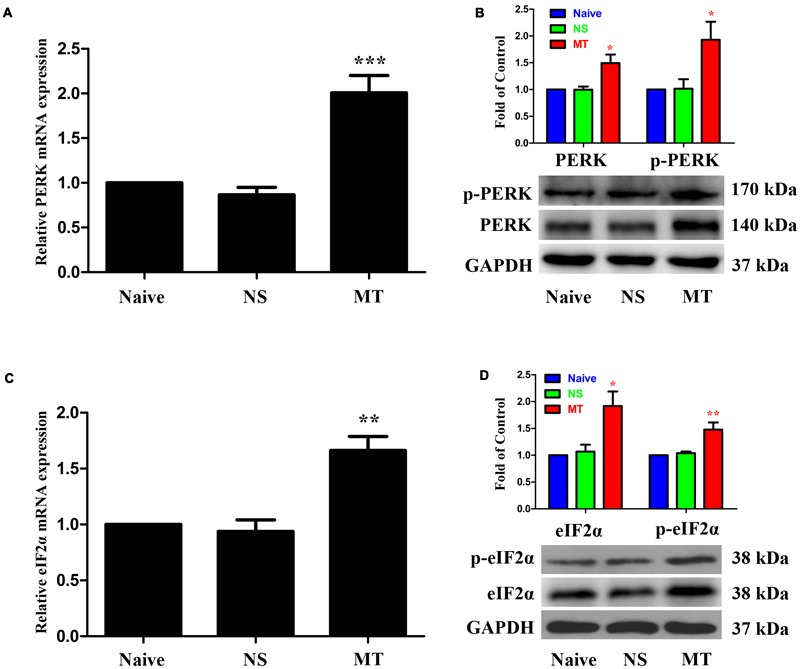
Expressions of PERK/eIF2α cascade in spinal cord. The expressions of PERK **(A,B)**, p-PERK (B), eIF2α **(C,D)** and p-eIF2α **(D)** were significantly increased in morphine-tolerant rats measured by real-time PCR and western blots, respectively. (^∗^*p* < 0.05, ^∗∗^*p* < 0.01, ^∗∗∗^*p* < 0.001 compared with naive rats, *n* = 4 in each group) PERK: protein kinase RNA-like ER kinase. p-PERK: phosphorylation of PERK. eIF2α, eukaryotic initiation factor 2 subunit alpha; p-eIF2α, phosphorylation of eIF2α; NS, normal saline; MT, morphine tolerance.

**FIGURE 6:**
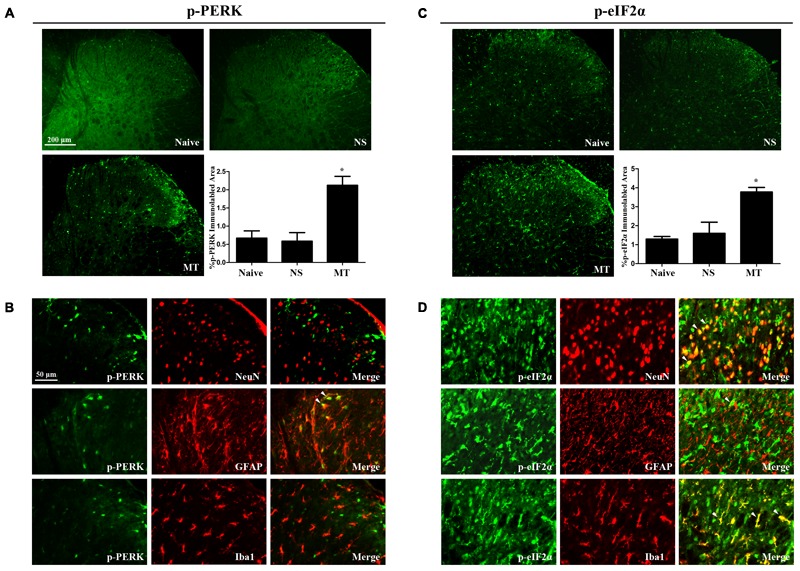
Distributions and cellular localizations of p-PERK and p-eIF2α in spinal cord. **(A,C)** Immunostaining of p-PERK and p-eIF2α in spinal dorsal horn. P-PERK and p-eIF2α were extensively expressed in spinal dorsal horn. The expressions of p-PERK and p-eIF2α were significantly increased in morphine-tolerant rats compared with those in naïve rats, respectively. (^∗^*p* < 0.05 compared with naive rats, *n* = 3 in each group, scale bar: 200 μm). **(B,D)** Double immunostaining of p-PERK and p-eIF2α and cell-specific markers in morphine-tolerant rats. The p-PERK was co-localized with GFAP (indicated by arrows), not with NeuN and Iba1. And p-eIF2α was mainly colocalized with NeuN and Iba1 (indicated by arrows), and a minority with GFAP. Scale bar: 50 μm. PERK, protein kinase RNA-like ER kinase; p-PERK, phosphorylation of PERK; eIF2α, eukaryotic initiation factor 2 subunit alpha; p-eIF2α, phosphorylation of eIF2α; NS, normal saline; MT, morphine tolerance.

To investigate the expressions of PERK/eIF2α cascade during the development of morphine tolerance, rats were treated with morphine or saline for 7 days. And the mRNA levels of PERK (**Figure 5A**, *F*_2,11_ = 26.03, *p* < 0.001) and eIF2α (**Figure 5C**, *F*_2,11_ = 18.64, *p* < 0.01) were detected to be increased in morphine-treated rats. Consistently, the protein levels of PERK (*F*_2,11_ = 8.539) and eIF2α (*F*_2,11_ = 8.66) were also increased in morphine-treated rats (**Figures 5B,D**, *p* < 0.05), as well as the p-PERK (**Figure 5B**, *F*_2,11_ = 5.875, *p* < 0.05) and eIF2α (p-eIF2α) (**Figure 5D**, *F*_2,11_ = 11.21, *p* < 0.05). The results of immunofluorescent staining also confirmed the increased immunoreactivity of p-PERK (**Figure 6A**, *F*_2,8_ = 14.43, *p* < 0.05) and p-eIF2α (**Figure 6C**, *F*_2,8_ = 13.05, *p* < 0.05) in spinal dorsal horn induced by chronic morphine treatment. To determine the cellular localizations of p-PERK and p-eIF2α in spinal cord, double immunofluorescent staining was performed in morphine-treated rats. The results showed that p-PERK was co-localized with GFAP (**Figure 6B**), while p-eIF2α was mainly co-localized with NeuN and Iba1, and a minority with GFAP (**Figure 6D**). These results indicate that chronic morphine treatment could result in the activation of PERK/eIF2α cascade which may play a fundamental role in neuron-glia and astrocyte-microglia interactions in the mechanism of morphine tolerance.

**FIGURE 7:**
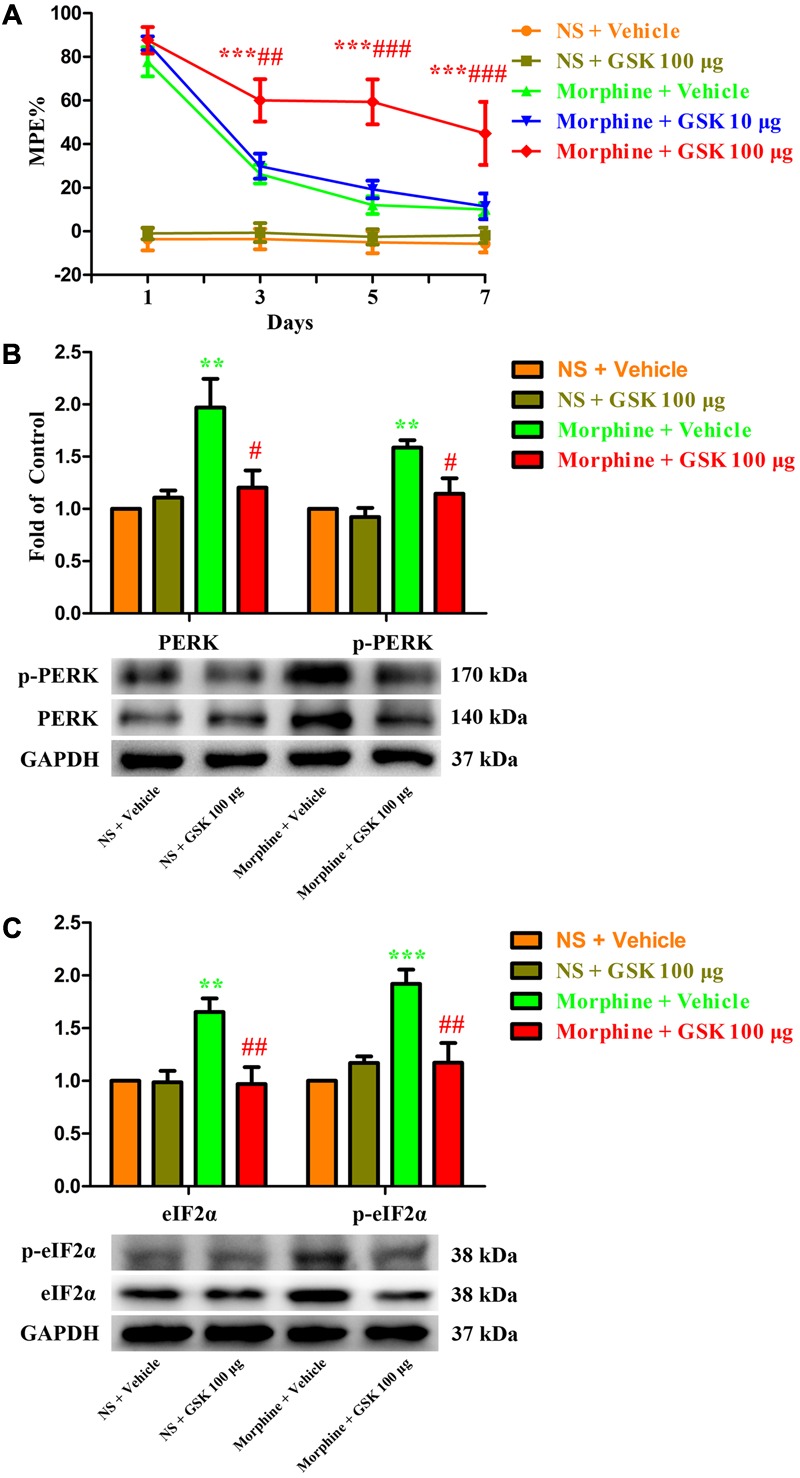
Effect of PERK inhibitor on the development of morphine tolerance. Specific PERK inhibitor GSK2606414 (10 μg or 100 μg) was intrathecal injected 30 min before morphine treatment for 7 days, respectively. **(A)** Pretreatment with 100 μg GSK2606414 could attenuate the development of morphine tolerance. (^∗∗∗^*p* < 0.001 compared with morphine + vehicle group; ^##^*p* < 0.01, ^###^*p* < 0.001 compared with morphine + GSK 10 μg group, *n* = 6 in each group). **(B,C)** Pretreatment with 100 μg GSK2606414 could inhibit the up-regulation of PERK, p-PERK **(B)**, eIF2α and p-eIF2α **(C)** induced by chronic morphine treatment measured by western blots. (^∗∗^*p* < 0.01, ^∗∗∗^*p* < 0.001 compared with NS + vehicle group; ^#^*p* < 0.05, ^##^*p* < 0.01 compared with morphine + vehicle group, *n* = 6 in each group). %MPE, percentage of maximal possible antinociceptive effect; GSK, GSK2606414; PERK, protein kinase RNA-like ER kinase; p-PERK, phosphorylation of PERK; eIF2α, eukaryotic initiation factor 2 subunit alpha; p-eIF2α, phosphorylation of eIF2α; NS, normal saline.

To further investigate the role of spinal PERK/eIF2α cascade in the development of morphine tolerance, a specific PERK inhibitor GSK2606414 was intrathecal injected 30 min before morphine administration. As shown in **Figure 7A**, a single dose of GSK2606414 (10 μg or 100 μg) did not affect the antinociceptive effect of morphine on day 1 of morphine administration (*p* > 0.05), but consecutive treatments with GSK2606414 (100 μg, but not 10 μg) could significantly attenuate the development of morphine tolerance from days 3 to 7 (*F*_1,30_ = 1.639, for 10 μg GSK2606414, *p* > 0.05; *F*_1,30_ = 11.24, for 100 μg GSK2606414, *p* < 0.05). Moreover, the increased expressions of PERK, p-PERK, eIF2α, and p-eIF2α induced by chronic morphine treatment could be inhibited by GSK2606414 pretreatment (**Figures 7B,C**, *F*_3,23_ = 7.225, for protein of PERK, *F*_3,23_ = 10.04 for protein of p-PERK, *p* < 0.05; *F*_3,23_ = 8.26, for protein of eIF2α, *F*_3,23_ = 11.84 for protein of p- eIF2α, *p* < 0.05). Taken together, these results suggest that PERK/eIF2α cascade might be involved in the development of morphine tolerance via mediating interactions between neural cells in spinal cord.

### Spinal ATF6 Contributed to the Development of Morphine Tolerance

**FIGURE 8:**
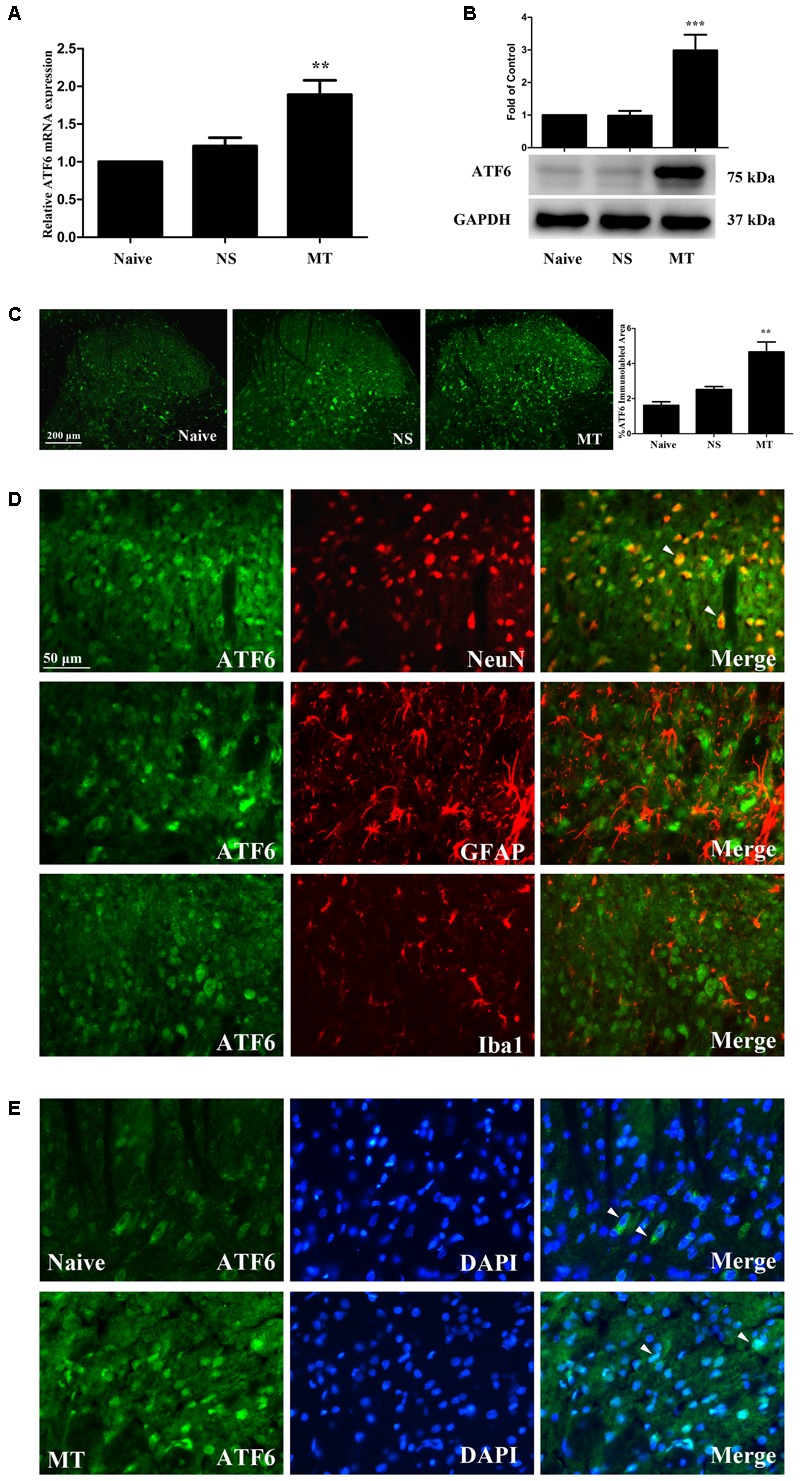
Expression of ATF6 in spinal cord. **(A,B)** The expression of ATF6 were significantly increased in morphine-tolerant rats measured by real-time PCR and western blots, respectively. (^∗∗^*p* < 0.01, ^∗∗∗^*p* < 0.001 compared with naive rats, Real-time PCR: *n* = 4 in each group; western blots: *n* = 6 in each group). **(C)** Immunostaining of ATF6 in spinal dorsal horn. ATF6 was extensively expressed in spinal dorsal horn. The immunoreactivity of ATF6 was significantly increased in morphine-tolerant rats. (^∗∗^*p* < 0.01 compared with naive rats, *n* = 3 in each group, scale bar: 200 μm). **(D)** Double immunostaining of ATF6 and cell-specific markers in morphine-tolerant rats. ATF6 was co-localized with NeuN (indicated by arrows), not with GFAP or Iba1 in spinal dorsal horn. Scale bar: 50 μm. **(E)** Chronic morphine administration results in ATF6 translocation to the nucleus. ATF6 was only expressed in cytoplasm in naive rats (indicated by arrows), while ATF6 was expressed in cytoplasm and cell nucleus in morphine-tolerant rats (indicated by arrows). Scale bar: 50 μm. ATF6, activating transcription factor 6; NS, normal saline; MT, morphine tolerance.

To investigate the expression of ATF6 during the development of morphine tolerance, rats were treated with morphine or saline for 7 days. And both the mRNA (**Figure 8A**, *F*_2,11_ = 13.57, *p* < 0.01) and protein (**Figure 8B**, *F*_2,17_ = 15.83, *p* < 0.001) levels of ATF6 was increased in morphine-treated rats. The results of immunofluorescent staining also confirmed the increased immunoreactivity of ATF6 (**Figure 8C**, *F*_2,8_ = 18.54, *p* < 0.01) in spinal dorsal horn induced by chronic morphine treatment. To determine the cellular localization of ATF6 in spinal cord, double immunofluorescent staining was performed in morphine-treated rats. The results showed that ATF6 was co-localized with NeuN, not with GFAP or Iba1 (**Figure 8D**). As a transcription factor, ATF6 was found to be translocated from cytoplasm to nucleus after chronic morphine treatment, while it was only expressed in cytoplasm in naive rats (**Figure 8E**). These results indicate that chronic morphine treatment result in the activation of ATF6 in neurons in spinal cord and promote the translocation of ATF6 to nucleus where it could induce the transcription of certain molecules.

**FIGURE 9:**
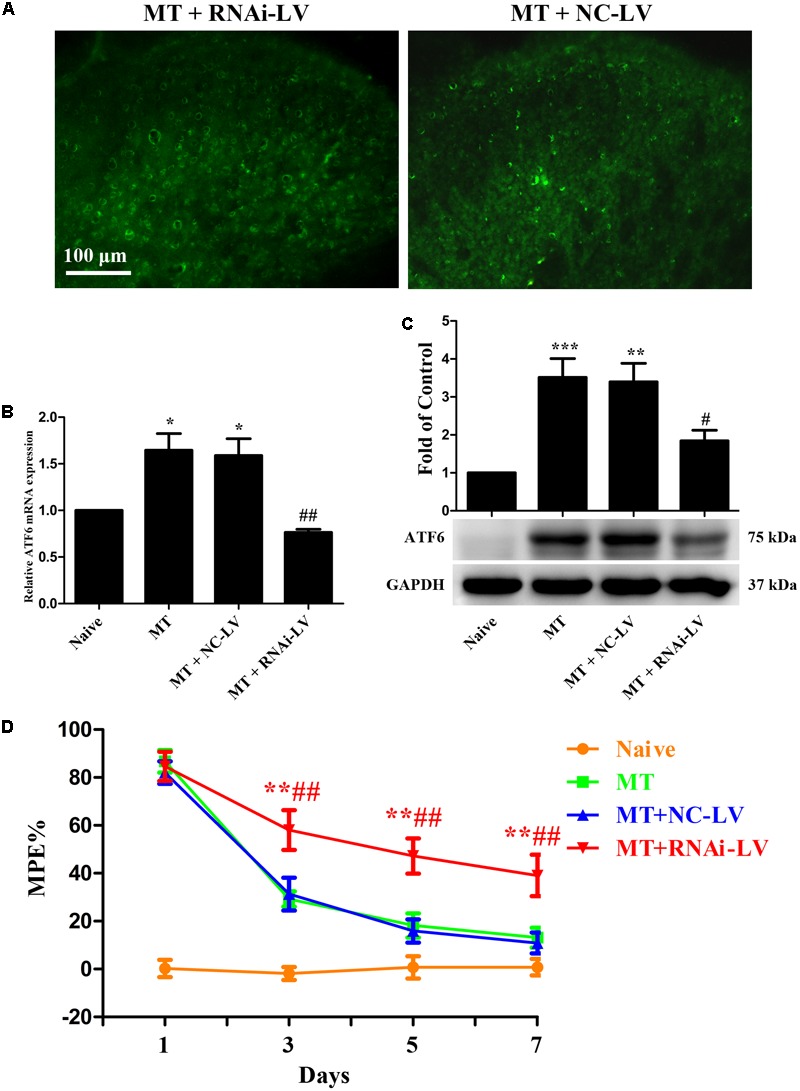
Effect of ATF6 RNAi-Lentivirus on the development of morphine tolerance. **(A)** Detection of lentivirus transfection in spinal cord. RNAi-LV or NC-LV was injected into lumbar spinal cord 3 days before intrathecal catheterization, respectively. The expression of enhanced green fluorescent protein in lentiviral vectors was detected in spinal dorsal horn *n* = 3 in each group. Scale bar: 100 μm. **(B,C)** RNAi-LV could effectively downregulate the expression of ATF6 induced by chronic morphine treatment measured by real-time PCR and western blots, respectively. (^∗^*p* < 0.05, ^∗∗^*p* < 0.01, ^∗∗∗^*p* < 0.001 compared with naive rats, ^#^*p* < 0.05, ^##^*p* < 0.01 compared with MT+NC-LV group, Real-time PCR: *n* = 4 in each group; western blots: *n* = 6 in each group). **(D)** Pretreatment with RNAi-LV could attenuate the development of morphine tolerance. (^∗∗^*p* < 0.01 compared with MT group; ^##^*p* < 0.01 compared with MT+NC-LV group, *n* = 6–8 in each group). NS, normal saline; MT, morphine tolerance; ATF6, activating transcription factor 6; %MPE, percentage of maximal possible antinociceptive effect.

To further investigate the role of ATF6 in the development of morphine tolerance, a recombinant shRNA lentiviral vector was used to knockdown the expression of ATF6 in spinal cord. 4 μL of RNAi-LV or NC-LV was injected into lumbar spinal cord of rats 3 days before intrathecal catheterization, respectively. The successful lentivirus transfection in spinal dorsal horn was verified by the expression of eGFP in RNAi-LV and NC-LV 1 week after microinjection (**Figure 9A**). The increased expressions of ATF6 in spinal cord, which were induced by chronic morphine treatment, could be inhibited by the transfection of RNAi-LV (**Figures 9B,C**, *F*_3,15_ = 11.59 for mRNA of ATF6, *F*_3,23_ = 10.69 for protein of ATF6, *p* < 0.05). The results of behavioral assessment demonstrated that transfection of RNAi-LV rather than NC-LV could significantly attenuate the development of morphine tolerance (**Figure 9D**, *p* < 0.01). Taken together, these results suggest that activation of neuronal ATF6 in spinal cord might be involved in the development of morphine tolerance.

## Discussion

Our study demonstrated the potential role of ER stress in spinal cord in the development of morphine tolerance. BiP, a marker of ER stress and standard indicator of UPR initiation, was up-regulated by chronic morphine treatment in spinal neurons. Three UPR pathways, including IRE1/XBP1 cascade, PERK/eIF2α cascade, and ATF6, were activated in different neuronal cells in spinal cord during the development of morphine tolerance. Importantly, inhibiting IRE1, PERK, or ATF6 could block UPR pathways and consequently attenuate the development of morphine tolerance, respectively. Taken together, our results provide the direct evidences that ER stress in spinal dorsal horn might be involved in the development of morphine tolerance.

Several mechanisms have been reported to underlie the development of morphine tolerance, including activation of cAMP pathway (Nestler and Aghajanian, 1997), NMDA receptors, CGRP receptors and neuronal GRs (Trujillo and Akil, 1991; Menard et al., 1996; Powell et al., 2000; Lim et al., 2005a,b), PKA, PKC, and MAPK (Mao et al., 1994; Cui et al., 2006; Chen et al., 2008; Wang et al., 2009). Recently, a unique phenomenon of morphine was discovered that morphine could alter the balance of excitatory and inhibitory synapses in hippocampus through a pathway involving the generation of reactive oxygen species and subsequent activation of ER stress which eventually initiate autophagy (Cai et al., 2016). ER is a highly dynamic organelle with multiple functions including protein folding and secretion, calcium homeostasis, and lipid biosynthesis (Hawes et al., 2015; Westrate et al., 2015). The physiological functions of ER stress is to induce the activation of UPR and clear unfolded/misfolded proteins and restore ER homeostasis (Rivas et al., 2015; Grootjans et al., 2016). However, excessive or unresolved ER stress inevitably lead to the activation of apoptotic pathways and cell death (Hetz, 2012). Previous studies have shown that ER stress and subsequent UPR were involved in obesity (Ozcan et al., 2004; Hotamisligil, 2005), heart disease (Yamaguchi et al., 2003), ischemia/reperfusion injury (Kumar et al., 2001), diabetes (Guerrero-Hernandez et al., 2014; Engin, 2016), atherosclerosis (Ivanova and Orekhov, 2016; Dong et al., 2017), cancer (Verfaillie et al., 2013; Clarke et al., 2014), Alzheimer’s disease and Parkinson’s disease (Dawson and Dawson, 2003; Li et al., 2015), as well as various types of chronic pain, including DPN (O’Brien et al., 2014), orofacial inflammatory pain (Yang E.S. et al., 2014), neuropathic pain (Inceoglu et al., 2015; Zhang et al., 2015). BiP is a central player in ER homeostasis. Under physiological conditions, BiP is associated with the ER stress sensors IRE1, PERK, and ATF6. When ER stress occurs, BiP dissociates from ER stress sensors and binds to misfolded proteins, resulting in the activation of three branches of UPR (O’Brien et al., 2014). Dobashi et al. (2010) reported that BiP, as an ER chaperone, could modulate the development of morphine antinociceptive tolerance. They tested the thermal antinociceptive effect of morphine in heterozygous mutant BiP mice and found that the mutant BiP mice showed less morphine tolerance. Furthermore, a chemical chaperone called Tauroursodeoxycholic acid which can improve ER protein folding capacity could promote the antinociceptive effect of morphine in wild-type mice, indicating that ER chaperone BiP plays a crucial role in morphine tolerance. In the present study, we provided the first evidence that the expression of BiP was up-regulated in neurons in spinal dorsal horn after chronic morphine treatment. This indicates that ER stress response and the occurrence of active UPR in spinal cord are closely related to the development of morphine tolerance.

Endoplasmic reticulum stress has been implicated to participate in the mechanism of neuropathic pain (Inceoglu et al., 2015; Zhang et al., 2015), inflammatory pain (Yang E.S. et al., 2014) and SCI in the spinal level (Ohri et al., 2011, 2013). In a rat model of L5 spinal nerve ligation (SNL)-induced neuropathic pain, the ER stress and UPR pathways were significantly activated (Zhang et al., 2015). Moreover, intrathecal administration of ATF6 siRNA attenuated neuropathic pain and BIP expression in the spinal cord. In another study, the activation of ER stress was detected in the peripheral nervous system of diabetic nephropathy rats (Inceoglu et al., 2015). Interestingly, chemical inducers of ER stress could lead to pain behavior which can be reversed by a chemical chaperone. An early activation of UPR pathways could be detected after SCI (Ohri et al., 2011, 2013). Moreover, SCI could lead to the up-regulation of several key ER stress response genes (e.g., ATF4, GADD34, GRP78, and CHOP) at the injury epicenter. Additionally, Genetic and pharmacological modulation of ER stress response via CHOP or PERK/eIF2α could improve the functional recovery after SCI. These studies provided solid evidence that ER stress play a pivotal role in chronic pain. In this study, we found the increased expressions of both spliced and XBP1u, which are related to IRE1 branch, PERK/eIF2α and their active forms, as well as ATF6 in spinal cord during the development of morphine tolerance. And pharmacological or genetic blockade of three UPR pathways could attenuate the development of morphine tolerance. So far, only a few studies have reported the effect of opioid on ER stress. *In vitro* study suggested that morphine could protect primary cultured astrocytes from glutamate-induced apoptosis via reducing Ca^2+^ overload and ER stress pathways (Zhang et al., 2016). Naloxone, an opioid receptor antagonist, could upregulate gene expression of ER chaperones in PC12 cells, including BiP, calnexin, ER protein 29 and protein disulfide isomerase, and ER stress sensors, including ATF6, IRE1, and PERK. In addition, naloxone could also induce typical ER stress phenomena, including ART6 proteolytic cleavage, eIF2α phosphorylation and XBP1 mRNA splicing (Seo et al., 2014). Jian et al. (2014) found that the retrieval of morphine memory was associated with the dephosphorylation of eIF2α expression in basolateral amygdala. Our results confirm the involvement of ER stress including three sensors and related signaling pathways in the development of morphine tolerance. Although blocking either one of three UPR branches could partially prevent the decline of morphine analgesic effect after repeated administration, weight of the pathways in the mechanism of morphine tolerance remains unknown which is hard to determinate. We hypothesized that three branches of UPR may share synergistic effect in the development of morphine tolerance. Therefore, further research is required to explore the complex roles of ER stress in morphine tolerance.

Our previous studies have demonstrated the important role and complexity of neuron-glia interaction in the mechanism of morphine tolerance (Peng et al., 2017; Wang et al., 2017). In the present study, expressions of IRE1 and XBP1 were found to be increased mainly in neurons and astrocytes in spinal dorsal horn, while ATF6 expression was increased mainly in spinal neurons in morphine-tolerant rats. In supraspinal mechanisms of CNS disorders, the increased expressions of IRE1, XBP1, and ATF6 could be observed in neurons and astrocytes in different brain regions, which indicate the interaction between neurons and glia (Zhang et al., 2012; Yang W. et al., 2014; Wen et al., 2016; Duran-Aniotz et al., 2017). Our results imply that neurons might be specifically responsible for pain signaling in cooperation with ER stress response. Additionally, in our study, p-PERK was found to be activated mainly in astrocytes of the spinal dorsal horn in morphine-tolerant rats, while p-eIF2α mainly in neurons and microglia, and only a minority of p-eIF2α in astrocytes. Our results indicated that PERK/eIF2α cascade might play a fundamental role in neuron-glia and astrocyte-microglia interactions during the development of morphine tolerance. Previous study reported that PERK-eIF2α pathway was activated in astrocytes of the spinal dorsal horn in SNL model (Zhang et al., 2015). Phosphorylated eIF2α could be induced in spinal interneurons in a rabbit spinal cord ischemia model (Yamauchi et al., 2007). Therefore, PERK-eIF2α pathway may take part in the different pathophysiological processes through different patterns. We speculate that there may be a different function of PERK-eIF2α pathway in morphine tolerance compared to IRE1-XBP1 and ATF6 pathways. Thus, further research about PERK-eIF2α pathway in morphine tolerance is worthy to do.

In summary, our study provides the first direct evidences that ER stress might be a significant driver of the development of morphine tolerance in spinal cord. These findings implicate a potential therapeutic strategy to restore the antinociceptive effect of morphine by inhibiting ER stress sensors IRE1, PERK, and ATF6, which may contribute to expanding the morphine usage in clinical practice.

## Author Contributions

FG supervised the entire project, designed the research, and wrote the paper. DL wrote and edited the manuscript and conceived and designed the ideas. YZ, YP, PS, ZL, and QX interpreted and analyzed the data. YT, XT, HY, ZW, and WM made substantial, direct, and intellectual contribution to the work. All authors read and approved the final manuscript.

## Conflict of Interest Statement

The authors declare that the research was conducted in the absence of any commercial or financial relationships that could be construed as a potential conflict of interest.

## Abbreviations

ATF6: activating transcription factor 6
BiP: binding immunoglobulin protein
cAMP: adenosine 3′,5′-monophosphate
CGRP: calcitonin gene-related peptide
DPN: diabetic peripheral neuropathy
eGFP: enhanced green fluorescent protein
eIF2α: eukaryotic initiation factor 2 subunit alpha
ER: endoplasmic reticulum
GAPDH: glyceraldehyde-3-phosphate dehydrogenase
GFAP: glial fibrillary acidic protein
GRs: glucocorticoid receptors
HRP: horseradish peroxidase
Iba1: ionized calcium-binding adapter molecule 1
IRE1: inositol-requiring enzyme 1
MAPK: mitogen-activated protein kinase
MPE: maximal possible antinociceptive effect
NMDA: *N*-methyl-D-aspartate
p-eIF2α: phosphorylation of eIF2α
PERK: protein kinase RNA-like ER kinase
PFA: paraformaldehyde
PKA: protein kinase A
PKC: protein kinase C
p-PERK: phosphorylation of PERK
PVDF: polyvinylidene fluoride
SCI: spinal cord injury
SDS-PAGE: sodium dodecyl sulfate-polyacrylamide gel electrophoresis
UPR: unfolded protein response
XBP1: X-box binding protein 1
XBP1s: spliced variant of XBP1
XBP1u: unspliced XBP1
